# Correction: Canonical Ru(ii) tris-polypyridyl complexes as confocal-compatible stains for lignified secondary-wall domains in *Arabidopsis thaliana* stems

**DOI:** 10.1039/d6ra90097f

**Published:** 2026-08-20

**Authors:** Ignacio Fuentes, Juan A. Fuentes, Alexander Carreño, Allan Cortés, Pablo Sepúlveda-Orellana, Susana Saez-Aguayo, Rubén Polanco, Evys Ancede-Gallardo, Ana Gabriel Suárez, Maria Carolina Otero, Luis Leyva-Parra, Angel A. Martí, Adrián A. Moreno

**Affiliations:** a Laboratorio de Genética y Patogénesis Bacteriana, Facultad de Ciencias de la Vida, Universidad Andres Bello República 330 Santiago 8370186 Chile; b Doctorado en Biotecnología, Facultad de Ciencias de la Vida, Universidad Andrés Bello República 330 Santiago 8370186 Chile; c Laboratory of Organometallic Synthesis, Departamento de Ciencias Químicas, Facultad de Ciencias Exactas, Universidad Andrés Bello República 330 Santiago 8370186 Chile alexander.carreno@unab.cl; d Centro de Investigación de Resiliencia a Pandemias, Facultad de Ciencias de la Vida e Instituto de Salud Pública, Universidad Andres Bello República 330 Santiago 8370186 Chile jfuentes@unab.cl; e Centro de Biotecnología Vegetal, Facultad de Ciencias de la Vida, Universidad Andrés Bello República 330 Santiago 8370186 Chile; f Escuela de Química y Farmacia, Facultad de Medicina, Universidad Andrés Bello República 252 Santiago 8370186 Chile; g Centro de Investigación Para el Diseño de Materiales (CEDEM), Facultad de Ciencias Exactas, Departamento de Ciencias Químicas, Universidad Andrés Bello Avenida República 275 Santiago 8370146 Chile; h Department of Chemistry, Rice University 6100 S. Main St Houston TX 77005 USA

## Abstract

Correction for ‘Canonical Ru(ii) tris-polypyridyl complexes as confocal-compatible stains for lignified secondary-wall domains in *Arabidopsis thaliana* stems’ by Ignacio Fuentes *et al.*, *RSC Adv.*, 2026, https://doi.org/10.1039/d6ra04630d.

The authors regret that the name of one of the authors (Maria Carolina Otero) was shown incorrectly in the original article. In addition, the author contributions were incorrectly given. The corrected author list and contributions are as shown herein.

The authors regret the omission of the following acknowledgement: “S. Saez Aguayo thanks Fondecyt Regular 1250930 and the authors thank Dr Dayán Páez-Hernández (UNAB) for providing access to computational facilities.”

The Royal Society of Chemistry apologises for these errors and any consequent inconvenience to authors and readers.

